# The Magical Activation of Left Amygdala when Reading Harry Potter: An fMRI Study on How Descriptions of Supra-Natural Events Entertain and Enchant

**DOI:** 10.1371/journal.pone.0118179

**Published:** 2015-02-11

**Authors:** Chun-Ting Hsu, Arthur M. Jacobs, Ulrike Altmann, Markus Conrad

**Affiliations:** 1 Department of Education and Psychology, Freie Universität Berlin, D-14195, Berlin, Germany; 2 Languages of Emotion, Freie Universität Berlin, D-14195, Berlin, Germany; 3 Dahlem Institute for Neuroimaging of Emotion (D.I.N.E.), Freie Universität Berlin, D-14195, Berlin, Germany; 4 Department of Cognitive, Social and Organizational Psychology, Universidad de La Laguna, 38205, San Cristóbal de La Laguna, Spain; Emory University, UNITED STATES

## Abstract

Literature containing supra-natural, or magical events has enchanted generations of readers. When reading narratives describing such events, readers mentally simulate a text world different from the real one. The corresponding violation of world-knowledge during this simulation likely increases cognitive processing demands for ongoing discourse integration, catches readers’ attention, and might thus contribute to the pleasure and deep emotional experience associated with ludic immersive reading. In the present study, we presented participants in an MR scanner with passages selected from the Harry Potter book series, half of which described magical events, while the other half served as control condition. Passages in both conditions were closely matched for relevant psycholinguistic variables including, e.g., emotional valence and arousal, passage-wise mean word imageability and frequency, and syntactic complexity. Post-hoc ratings showed that readers considered supra-natural contents more surprising and more strongly associated with reading pleasure than control passages. In the fMRI data, we found stronger neural activation for the supra-natural than the control condition in bilateral inferior frontal gyri, bilateral inferior parietal lobules, left fusiform gyrus, and left amygdala. The increased activation in the amygdala (part of the salience and emotion processing network) appears to be associated with feelings of surprise and the reading pleasure, which supra-natural events, full of novelty and unexpectedness, brought about. The involvement of bilateral inferior frontal gyri likely reflects higher cognitive processing demand due to world knowledge violations, whereas increased attention to supra-natural events is reflected in inferior frontal gyri and inferior parietal lobules that are part of the fronto-parietal attention network.

## Introduction

Literary reading brings pleasures that are unique and important to human beings [1–3]. Interestingly, part of these pleasures seems to get more intense with increasing distance between what we read about and everything we ever have or ever will experience in our real lives. Supra-natural, or magical events in fictional literature like fairy tales enchant readers from an early age on, though even the very young audience seems to be aware of the “trick”: With a property attribution task, Sharon and Woolley [4] found that 4- and 5-year-olds’ abilities to differentiate between properties of real (child, clown) versus fantastic (Santa, Fairy, Superman) entities were the same as in adults [5]. Exposure to magical contents has been shown to facilitate creativity in children [6]. The present study aimed to investigate the neural correlates of reading about magical events in fictional literary texts in adult readers. The representation of magical events can be considered quite a demanding or sophisticated case of meaning construction, because for the sake of discourse processing, the reader is required to accept something she or he knows to be impossible in the real world. On the other hand, fantasy literature, typically involving descriptions of such impossible events, represents a most successful literature genre. Here, we wanted to explore how the brain processes the emergence of magic in the virtual world a book creates in our mind.

From a theoretical perspective, discourse comprehension involves three different processing steps [7, 8]: 1) the construction of the *surface structure* of a text, which is a mental representation of the exact text read; 2) a *text-base representation*, which contains idea units explicitly stated in the text, including bridging inferences that help connecting consecutive clauses; and 3) a *situation model* of the text, in which the current linguistic input (i.e., the linguistic meaning of the sentence or paragraph being read) is integrated with both general world knowledge and the prior discourse context [9, 10]. Supra-natural events, or magical events in discourse involve world-knowledge anomalies, the processing of which should engage the left inferior frontal gyrus (LIFG): Hagoort’s [11] proposal that world knowledge integration takes place in LIFG is indeed supported by studies suggesting that world knowledge anomalies caused stronger activation in LIFG [12], or bilateral IFG [13], as does meaning making with novel metaphors [14] and processing of figurative language in general [15].

Besides such specific responses to evident world-knowledge violations, readers seem to generally adopt different reading strategies for fictional as compared to factual texts: Unlike reading texts that merely describe certain events or facts, reading fiction involves processes of constructive content imagination and simulation [16], especially perspective taking and relational inferences [17] that bring enjoyment specific to fictional reading. Altmann et al. [18, 19] could show that respective differences in brain activation already emerge at a paratextual level depending only on whether readers believe a text to be factual or fictional: They presented short stories containing negatively valenced plots (crimes, disasters, accidents) and short neutral stories to participants after telling them that they were either reading invented (fictional) or real (factual) stories. Results showed that reading attitude or strategy for fictional texts was correlated with brain activity in right lateral frontopolar cortex associated with constructive content simulation [20], as well as in posterior cingulate cortex (PCC) and left precuneus related to Theory of Mind (ToM) and affective empathy [16, 21]. The effective connectivity between right lateral FPC and ToM related medial prefrontal cortex (mPFC), PCC, precuneus, and anterior temporal lobe (aTL) suggests that perspective taking and relational inferences [17] are required for the simulation processes when reading about events we consider as fictional.

In this study, we presented participants with entire text passages from a popular book series that has enchanted numerous readers across the globe – Harry Potter – to explore the neural correlates of processing supra-natural events in the context of fantasy literature – an extremely popular literature genre, whose enormous success asks for scientific explanations, including the neuronal level.

Harry Potter combines in an exemplary way the different textual aspects mentioned above that might constitute the specific fascinating experience inherent to fantasy literature: the unmistakable awareness of the fictional text character (due to the perfectly known protagonist Harry Potter) is likely to put readers in a predisposition for increased mental simulation [18, 19]. Furthermore, descriptions of magical events violate world-knowledge, catch the attention of readers [22], and bring about the novelty and emotional richness associated with the affective and aesthetic pleasure often characteristic of literary reading [23–26].

Based on previous results [12, 13] and a recent neurocognitive model of literary reading [25–27], we propose that the violation of world knowledge contained in described supra-natural events should increase the cognitive demand of world knowledge integration, and that the related novelty, unexpectedness, and uncertainty should activate the salience/emotion network [28, 29], which should further recruit the fronto-parietal attention networks [22, 30, 31]. Due to the limited temporal resolution of fMRI, we do not intend to disentangle different processes taking place in a sequence. We rather expect to reveal conjoint neural correlates of three processes related to the mental simulation of supra-natural events in the following neural substrates: 1) the knowledge-integration network, with the IFG as the core region [11–13]; 2) the salience network [28], mainly including OFC, dorsal anterior cingulate, anterior insula, amygdala, and other subcortical structures which are also highly associated with emotion processing [29], particularly the amygdala [32]; and 3) the fronto-parietal attention network, including temporo-parietal junction (TPJ), inferior parietal lobule (IPL), ventral frontal cortex including middle frontal gyrus, IFG, frontal operculum, and anterior insula [30, 31, 33].

## Materials and Methods

### 1. Stimuli

We selected twenty passages describing supra-natural events for the Supra-natural condition, e.g., “She waved her wand over her shoulder; a loaf of bread and a knife soared gracefully on to the table. As the loaf sliced itself and the soup pot dropped back on to the stove, Mrs Weasley sat down opposite him.”, and twenty passages devoid of supra-natural events, e.g., “Harry, Ron and Hermione descended Professor Trelawney's ladder and the winding staircase in silence, then set off for Professor McGonagall's Transfiguration lesson. It took them so long to find her classroom that, early as they had left Divination, they were only just in time.” for the Control condition from all seven Harry Potter (HP) novels [34–40] and the authorized German translations (by Klaus Fritz, Carlsen Verlag, Hamburg).

To avoid habituation to magic situations, stimuli were embedded in a total of 120 text passages that overall provided a wide range of emotional content typical for this type of literature. Beyond the topic of the present study, the entire stimulus material also served to address a research question related to bilingual language processing in highly proficient German-English bilinguals (see [41, 42] for details). Therefore, materials were presented in either the German or the English version balancing presented language across experimental conditions.

We ensured that understanding the passages did not require a high level of familiarity with HP novels.

Between the Supra-natural and Control conditions we matched the following potentially confounding factors: 1) emotional content, operationalized in valence [*F_(1,38)_* = 0.008] and arousal [*F_(1,38)_* = 0.27] ratings collected in a pilot study from 15 German native speakers for the authorized German translations; 2) narrative complexity, operationalized as the number of persons or characters (as a discrete variable, Pearson chi-square = 3.74; as a continuous variable, *F_(1,38)_* = 0.08), and the type of inter-character interaction (as a discrete variable, Pearson chi-square = 0); 3) passage length, operationalized in numbers of letters [*F_(1,38)_* = 1.36], words [*F_(1,38)_* = 1.66], sentences [*F_(1,38)_* = 0.08], and subordinate sentences [*F_(1,38)_* = 0.19] per passage across conditions; 4) imageability and frequency of presented concepts: passage-wise average word imageability [*F_(1,38)_* = 0.0001] taken from the Berlin Affective Word List reloaded (BAWL-R; [43]) and the Affective Norms for German Sentiment Terms (ANGST; [44]), and passage-wise average frequency of words given in SUBTLEX [log frequency *F_(1,38)_* = 1.16, p = 0.29] (Brysbaert et al., 2011), and Leipzig Wortschatz Lexicon [log frequency *F_(1,38)_* = 1.70, p = 0.20] (available at http://wortschatz.uni-leipzig.de/). The descriptive statistics of matched continuous variables are listed in Table 1.

**Table 1:** Balanced Emotional and Psycholinguistic Variables across Conditions.

		Means ± S.D. in Conditions	
	*F(1,38*)	Supra-natural	Control
N. of Letters	1.36	250.65 ± 37.76	235.75 ± 42.93
N. of Words	1.66	41.55 ± 7.32	38.63 ± 7.06
N. of Sentences	0.08	2.55 ± 0.97	2.45 ± 1.27
N. of Subordinates	0.19	5.95 ± 1.60	6.20 ± 1.96
N. of Persons	0.08	2.40 ± 0.94	2.30 ± 1.22
Valence Rating	0.008	-0.32 ± 1.78	-0.38 ± 2.10
Arousal Rating	0.27	3.30 ± 0.85	3.45 ± 0.94
Imageability	0.0001	4.46 ± 0.38	4.46 ± 0.46
Log-SUBTLEX	1.16	2.76 ± 0.35	2.88 ± 0.32
Log-Leipzig Freq.	1.70	0.84 ± 0.47	0.68 ± 0.26

### 2. Ethical Statement

All participants have given written consent to take part in the experiment, which was approved of by the ethics committee of Freie Universität Berlin, and conducted in compliance with the Code of Ethics of the World Medical Association (Declaration of Helsinki). Participants were compensated properly monetarily or with course credits for their participation.

### 3. Participants

Twenty-three right-handed native German speakers (sixteen female) took part in the experiment. Their age ranged from 19 to 31 (mean ± *SD* = 23.78 ± 3.73). All participants all had read at least one HP book, and had no problem understanding the novel-specific contents in German or English. All of them had normal or corrected-to-normal vision, and reported no neurological or psychiatric disorder.

### 4. Design and Procedure

A repeated measures design was applied with supra-naturality (“Supra-natural” and “Control”) as the within subject factor. We divided the passages into two subsets, each containing 10 supra-natural, 10 control, and 40 filler text passages. During the experiment, each participant read one subset, i.e. half of the passages in each condition, in German, and the rest in English.

The experiment consisted of four runs, each containing five supra-natural, five control, and 20 filler passages. We pseudo-randomized the assignment of specific passages to specific runs, the order of presentation of the 30 passages in each run and the order of runs per participant. Each participant had his or her own pseudo-randomization sequence of stimuli presentation, and all passages were presented evenly across different runs for all participants. For each participant, the distribution of the presented language in conditions and runs was balanced, i.e., we ensured that runs with three German supra-natural, two German control, and 10 German filler passages were always followed by runs with two, three, and 10 respective German passages. Similar to the design of a successful previous text-reading fMRI experiment [19], in the MR scanner each passage was presented for 14 s, distributed on four lines (shown 3.5 s each), and then followed by 14 s of fixation cross. After the last fixation block and until the end of the scanning, the participant was presented with a message that this run of experiment had ended and the staff will talk to the participant soon afterwards. The visual information was presented on a computer screen and was reflected to the participants’ eyes via a mirror.

To keep participants attentive, four randomly selected passages in each run were immediately followed by an emotion-unrelated, context-specific yes/no question (e.g. ‘Was Harry in a train station?’ ‘Was the alarm clock broken again?’), to which participants responded via button press (‘yes’, ‘no’). Each question was presented for four seconds, which included the time for response.

### 5. fMRI data acquisition

Functional data were acquired on a Siemens Tim Trio 3T MR scanner. Four runs of 440 volumes were measured using a $T_{2}^{*}$-weighted echo-planar sequence [slice thickness: 3 mm, no gap, 37 slices, repetition time (TR): 2s, echo time (TE): 30ms, flip angle: 70°, matrix: 64 × 64, field of view (FOV): 192 mm, voxel size: 3.0 mm × 3.0 mm × 3.0 mm] and individual high-resolution T1- weighted anatomical data (MPRAGE sequence) were acquired (TR: 1.9, TE: 2.52, FOV: 256, matrix: 256 × 256, sagittal plane, slice thickness: 1 mm, 176 slices, resolution: 1.0 mm × 1.0 mm × 1.0 mm).

### 6. fMRI preprocessing

The fMRI data were preprocessed and analyzed using the software package SPM8 (www.fil.ion.ucl.ac.uk/spm). Preprocessing consisted of slice-timing correction, realignment for motion correction, and sequential coregistration. Structural images were segmented into grey matter, white matter, cerebrospinal fluid, bone, soft tissue, and air/background with the ‘New Segment’ module [45]. A group anatomical template was created with DARTEL (Diffeomorphic Anatomical Registration using Exponentiated Lie algebra) [46] toolbox from the segmented grey and white matter images. Transformation parameters for structural images were then applied to functional images to normalize them to the brain template of the Montreal Neurological Institute (MNI) supplied with SPM. Functional images were resampled to a resolution of 1.5 × 1.5 × 1.5 mm, and spatially smoothed with a kernel of 6 mm full-width-at-half-maximum during normalization.

### 7. Post-hoc ratings

To address the impact of supra-natural text content at a behavioral level, we asked 20 native German speakers (12 female, age 18–54 years, mean age ± *SD* = 30 ± 10.08) who were rewarded with course credits to evaluate their subjective reading experience for our stimuli on four dimensions. Participants, again, all liked the Harry Potter novel series and had no problem understanding the passages. They were asked to rate each passage in the authorized German translation on the following dimensions scaled from 1 (not at all) to 7 (extremely): 1) *supra-naturalness*: “Does the text describe something supra-natural?”; 2) *surprise*: “Does the text passage contain surprising elements?”; 3) *reading pleasure*: “How much reading pleasure did the passage bring you?”.

The questionnaire was created with SoSci Survey [47] and made available to the participants on www.soscisurvey.com. We calculated mean rating values for each passage on each dimension for further analyses.

### 8. fMRI GLM Analyses

We calculated statistical parametric maps by multiple regressions of the data onto a model of the hemodynamic response [48]. In the subject-level analysis, this model contained regressors for the passage onsets for each of the three experimental conditions (*Supra-natural, Control, fillers*). The duration for each passage was 14 seconds. The context-specific questions were modeled as the fourth condition, and each question lasted four seconds. The fourth condition also modeled the final message between the end of the experiment and the end of the scanning. The six realignment parameters were modeled as six additional regressors. Regressors were convolved with the canonical hemodynamic response function in SPM8. A temporal high-pass filter with a cutoff of 128 s was applied. Contrasts of the [*Supra-natural*] and [*Control*] conditions for each participant were used at the group level to model a random effect one-way ANOVA. A conjunction contrast of [*Supra-natural >* fixation] and [*Control >* fixation] contrasts was made in the ANOVA analysis to show the neural correlates of fictional reading in this study. Contrasts of the [*Supra-natural*] and [*Control*] conditions for each participant were used at the group level to model a random effect paired t-test analysis, to show the differential activation between two experimental conditions.

The fMRI analyses were conducted at the whole brain level. However, numerous previous studies and a connectivity study on the salience network [28] strongly suggested that salience is a key driver for amygdala activity [29, 49–51]. Recent meta-analyses also strongly suggest the amygdala to be involved in emotion processing [29, 32, 52, 53], especially in emotional discourse comprehension [54]. Therefore, we performed small volume correction (SVC) with a bilateral amygdala mask for contrasts comparing Supra-natural vs. Control conditions. The bilateral amygdala mask in the MNI template was defined by the WFU Pickatlas Tool [55].

For whole-brain fMRI analyses, we used an initial voxel-level threshold of uncorrected p < 0.005, then a cluster-level threshold of false-discovery rate (FDR) corrected p < 0.05 for the entire image volume, as suggested by Liebermann and Cunningham [56] for studies in cognitive, social and affective neuroscience. For the SVC analyses of amygdala, we used initial voxel-level threshold of uncorrected p < 0.005 for the entire image volume, then the threshold of voxel-level family-wise error (FWE) corrected p < 0.05 after applying the SVC with a bilateral amygdala mask. The labels reported were taken from the ‘TD Labels’ [57, 58] or ‘aal’ labels in the WFU Pickatlas Tool. The Brodmann areas (BA) were further checked with the Talairach Client using nearest grey matter search after coordinate transformation with the WFU Pickatlas Tool.

## Results

### 1. Behavioral performance

All 23 participants correctly responded to context-specific questions in the scanner above chance (≥ 50%) with overall mean accuracy of 83.12% ± 10.51%. One-tailed t-test against chance level was highly significant (*t* = 37.71, *p* ≤ 0.0001)

### 2. Post-scan emotion ratings: valence and arousal

Post-scan ratings by 23 participants of the fMRI experiment confirmed the balance of items concerning these classical emotion dimensions across Supra-natural vs. Control conditions (valence, *F_(1,38)_* = 0.04; arousal, *F_(1,38)_* = 0.99).

### 3. Post-hoc rating effects on additional evaluation dimensions

Mean ratings for the dimensions of supra-naturalness, surprise, and reading pleasure were significantly higher in the Supra-natural than the Control condition (Table 2) according to Student’s t-tests.

**Table 2:** Student’s t-tests of Post-hoc Ratings Across Conditions.

			Mean Values ± S.D.	
	t(38)	p-value	Supra-natural	Control
Supra-naturalness	6.13	<.0001	5.48 ± 0.93	3.19 ± 1.38
Surprise	3.69	0.0007	4.14 ± 0.76	3.18 ± 0.88
Reading Pleasure	3.01	0.0046	4.42 ± 0.36	4.09 ± 0.34

### 4. fMRI Results

In the conjunction of the two conditions [*Supra-natural* > fixation] and [*Control* > fixation], we found the following neural correlates that were more active in both Supra-natural and Control conditions in comparison with the baseline (Table 3): bilateral striate and extrastriate visual cortex (BA 17 & 18), bilateral dmPFC (BA 6), right superior and middle temporal gyrus, from anterior to posterior temporal lobe (BA 21 & 22), bilateral medial paracentral gyrus (BA 6), bilateral OFC (BA 11).

**Table 3:** Results of the Conjunction Analysis for Supra-natural and Control Conditions.

H	Regions	Voxel	p	T	B.A.	[*x, y, z*]
Conjunction: [Supra-natural > fixation] & [Control > fixation]						
L+R	Occipital pole (lingual gyrus)	98670	<.001	19.53	18/17	22 -87 -11
				18.33	17	-12 -97 -6
L+R	dmPFC (SFG, medial FG)	3282	<.001	16.37	6	-3 2 66
				7.46	6	8 12 49
R	STG, MTG	16161	<.001	13.20	22/21	50 -28 -0
L+R	Medial paracentral & precentral gyrus	1944	<.001	7.49	6	9 -33 58
				5.39	4/6	-8 -39 61
L+R	OFC (medial FG & orbital gyrus)	514	0.003	6.46	11	-4 47 -18
				5.30	11	3 44 -20

Abbreviations: H – hemisphere; L – left hemisphere; R – right hemisphere; T – t-values; B.A. – Brodmann area; dmPFC – dorsomedial prefrontal cortex; FG – frontal gyrus; SFG – superior frontal gyrus; MTG – middle temporal gyrus; OFC – orbitofrontal cortex; STG – superior temporal gyrus

**Fig 1:**
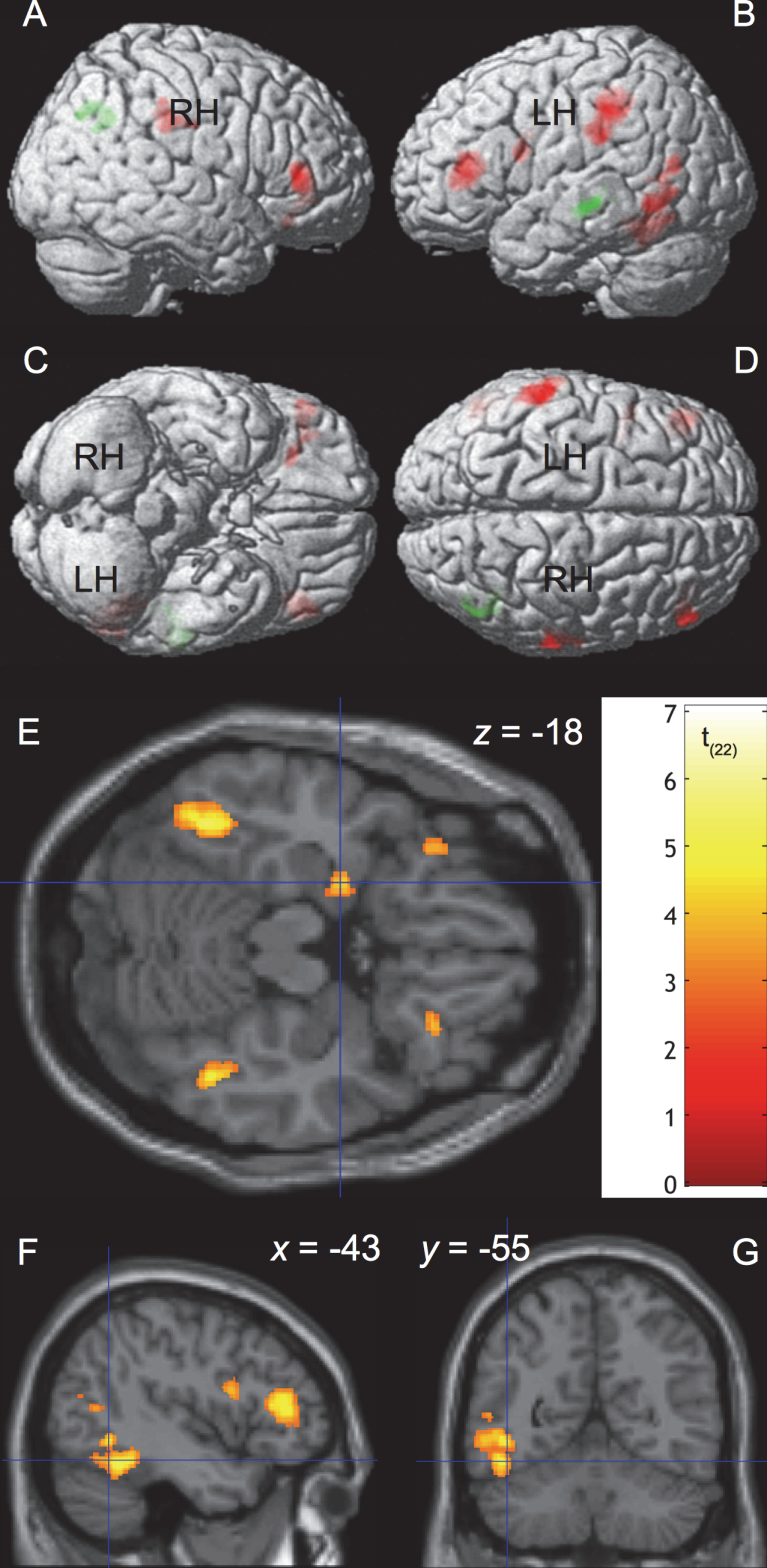
Effects of Supra-natural vs. Control Conditions. Regions showing significant BOLD response differences for supra-natural vs. control passages (Table 4)—created using xjView toolbox. Red color indicates significant positive differences (Supra-natural > Control). Green color indicates significant negative differences (Supra-natural < Control). A: right lateral view; B: left lateral view; C: inferior view; D: superior view; E: transverse section highlighting left amygdala in the contrast [Supra-natural > Control]. The color bar shows the t-value. Cross-hair: MNI coordinate -22 -3 -18. F and G: in the contrast [Supra-natural > Control], the cluster in the left fusiform gyrus contains the voxel (cross-hair: MNI -43 -55 -17; Talairach -43 -54 -12) reported as Visual Word Form Area (VWFA) by Cohen, et al. [65]. F: sagittal section. G: coronal section.

The results of paired t-tests are listed in Table 4 and shown in Fig. 1. In the contrast [*Supra-natural > Control*], the following neural correlates were significantly more active when participants read Supra-natural passages as compared to Control passages: two cluster in the LIFG pars triangularis (BA 46 & 13) and opercularis (BA 44 & 9), one cluster in the RIFG spanning pars triangularis and pars orbitalis (BA 45 & 47), bilateral TPJ spanning IPL and postcentral gyrus (BA 40, 3 & 2), and left fusiform gyrus (BA 37, Fig. 1A-D, red color, Fig. 1F). After applying the SVC, activation of left amygdala was found to be significantly increased for supra-natural passages (Fig. 1E).

**Table 4:** Results of the Paired t-test for Supra-natural vs. Control Conditions.

H	Regions	Voxel	p	T	B.A.	[*x, y, z*]
**Supra-natural > Control**						
R	IFG pars triangularis, orbitalis & MFG	801	<.001	7.04	45/47	50 38 6
L	Fusiform, ITG	1894	<.001	5.55	37	-48 -60 -14
L	TPJ (postcentral gyrus & IPL)	1002	<.001	5.48	2/40	-58 -33 40
L	IFG pars triangularis	886	<.001	5.14	46/13	-45 38 9
L	IFG pars opercularis	370	0.022	4.28	44/9	-51 11 19
R	TPJ (IPL & postcentral)	353	0.022	4.10	40/3/2	66 -31 33
L	Amygdala (SVC)	139	0.016	4.45		-22 -3 -18
**Supra-natural < Control**						
L	MTG	443	0.021	5.01	21/22	-64 -24 -8
R	Supramarginal, precuneus, IPL	442	0.021	4.09	40/39	40 -55 36

Abbreviations: H – hemisphere; L – left hemisphere; R – right hemisphere; T – t-values; B.A. – Brodmann area; FG – frontal gyrus; IFG – inferior frontal gyrus; IPL – inferior parietal lobule; ITG – inferior temporal gyrus; MFG – middle frontal gyrus; MTG – middle temporal gyrus; STG – superior temporal gyrus; SVC – small volume correction; TPJ – temporo-parietal junction

In the contrast [*Supra-natural < Control*], the following neural correlates were significantly less active when participants read supra-natural passages as compared to control passages: the left lateral temporal cortex (BA 21 & 22) and right IPL and supramarginal gyrus (BA 40 & 39, Fig. 1A-D, green color, Fig. 1G).

## Discussion

### 1. Effects of fictional Literature

For the conjunction of the conditions [*Supra-natural*] and [*Control*] results are in line with previous results for reading (emotion-laden) texts: bilateral medial paracentral gyrus (BA 6) is associated with encoding written language [59, 60]. Lateral temporal cortex, aTL and dmPFC are associated with multi-modal semantic integration [61, 62], and are part of the Extended Language Network (ELN) [60]. The impact of general emotionality of the passages is reflected in the activity of bilateral orbitofrontal gyrus (cf. [54, 63, 64]), and bilateral aTL, which is highly associated with emotional semantic processing [29, 62].

## 2. Effects of Supra-natural Events

We hypothesized that reading fiction involving supra-natural events increases the cognitive demands associated with world knowledge integration, activates the salience and emotion network, and recruits the fronto-parietal attention networks.

Our results support this hypothesis. Post-hoc ratings (Section 3.3) back-up the validity of our manipulation in terms of perceived supra-naturalness, but they also revealed important differences concerning the emotional appeal of our stimuli: despite being controlled for valence and arousal between Supra-natural and Control conditions, readers found passages with supra-natural events more surprising and found reading them more enjoyable than control passages.

In the neuroimaging data, we found 1) two clusters of activation in LIFG and one cluster in RIFG (exactly in BA 45 and 47) associated with increased demand of world knowledge integration [12, 13]; 2) increased activation in the left amygdala, which is part of the salience and the emotion networks [28, 29, 32]; 3) activation in bilateral IFG and IPL associated with increased attention recruited by the salience of supra-natural events in the fronto-parietal attention network [30, 31].

In addition, we found a cluster in the left fusiform gyrus, which contained the coordinate consistently reported as Visual Word Form Area (VWFA, transformed MNI coordinate -43 -55 -17, specified in Fig. 1F & G by the crosshair, cf. [65]) being more activated in the Supra-natural than in the Control condition. According to the interactive perspective of visual feature processing in the fusiform gyrus [66–69], increased VWFA activation could be either due to top-down influence depending on task demand or to bottom-up processing of visual features like string length, bigram frequency, total line length, or the number of line endings of words and pictures [67]. As we had balanced the number of letters and words across the Supra-natural and Control conditions, increased VWFA activation is unlikely to be driven by visual feature related bottom-up processing. We consider it, instead, to be top-down attention-driven, resulting from the effort to resolve the uncertainty or surprise due to the supra-natural events by keeping up the reading process [29].

As it is perhaps the most important among our findings, the increased activation of left amygdala when reading about magical events deserves detailed discussion: left lateralization of the amygdala activation was also observed in other studies using linguistic material as stimuli [70], and can be attributed to left hemispheric dominance for language processing [71, 72] and a sign for higher emotional relevance [73] and/or affective intensity [74] of figurative language [15, 75]. Several meta-analyses [32, 52, 53] have shown that the amygdala is traditionally associated with emotion processing. Many studies already suggested the amygdala to be generally sensitive to salient stimuli, including emotionally salient ones [29, 49–51], while Seeley et al. [28] identified the amygdala as part of the intrinsically connected salience network.

The theoretical framework of the neurocognitive model of literary reading [25, 26] offers an interesting account on how an interaction between salience and emotion processing could contribute to the specific experience of literary reading: Violation of world knowledge would activate the salience network, and once a text is accordingly processed as something that interestingly differs from everyday language use, the reader is set in a more receptive mood to perceive and enjoy the affective elements specific to the text (see [76] for fMRI data). We therefore tentatively propose the amygdala activation in the present data to be a function of salient features of magical events in a text. Considering the *surprise* and *reading pleasure* ratings for supra-natural text passages at the behavioral level (obtained from a different set of participants), it seems plausible that magical events are associated with the emotion of *surprise* [77] and the hedonic experience of *reading pleasure* [1–3]. Both phenomena may contribute to why common language use employs the term “magic” as a synonym of emotional intensity. Given that the amygdala has reliably been shown to be an integral part of the spread of activation over the brain processing emotional material, our finding that this key structure of emotion processing is activated by salient supra-natural text elements suggests that reading about events so charmingly beyond our everyday life experience lays the ground of gratifying emotional experiences associated with this literature. Note that our stimulus material features many highly emotional events in both conditions, but that the basic affective content of magical passages – as assessed by valence and arousal ratings – was kept comparable to those of the control condition. Certainly, our interpretation of the present amygdala activation involves some of the problems typical to reverse inference (but see [78], for a basic theoretical contribution on the issue), and amygdala activity in our data may also to some extent be accounted for by the mere salience of the unearthliness of magical passages. Taken together, we’d like to suggest thus that an intensified affective processing associated with supra-natural contents can be considered either an effect of discrete emotion [79–81] – e.g., surprise – not captured in the two-dimensional affective space [82, 83], or an interaction of affective processing and world-knowledge violation processing when updating a given situation model [9, 10].

Finally, we also found two clusters of neural correlates in which the activity was weaker when participants read supra-natural passages as compared to control passages. The left middle temporal cortex has been associated with more basic language perception as part of the ELN [60], while the cluster in the right parietal lobe could be associated with autobiographical memory [84, 85], working memory [86], semantic integration [61, 62], default network [87], or Theory of Mind as well as social cognition processing [88]. We would like to present the following tentative explanation for these effects: Considering the association with processing of autobiographical memory, reading about supra-natural events, in comparison with normal passages, may induce less episodic recollection of personal events from one’s own life – presumably simply because such events are by definition beyond our experience. It is also possible that texts from the Supra-natural condition impose higher cognitive demand and, thus, deactivating the default network.

On the other hand, the brain activation patterns observed here offer a tentative explanation of why we apparently enjoy exactly this facet of fantasy literature so much: our mental simulations of such supra-natural events occupy our attention network and surprise and entertain us more strongly than comparable fictional, but non-supra-natural literature. Moreover, they particularly activate one brain structure that is not only involved in detecting salient events (which magical events definitely are), but most intimately linked to emotional and hedonic experience (most people presumably are striving for when reading novels): the amygdala.

### 3. Limitations and Conclusion

In this study we investigated the neural correlates of reading about supra-natural, magical events. Within the limits of a revised reverse inference logic [78], our results can be interpreted as indicating that supra-natural events: 1) recruit greater activity in bilateral IFG associated with the integration of ongoing discourse and world knowledge information; 2) activate left amygdala, which is part of the salience network involving emotion processing and further recruit the fronto-parietal attention network and VWFA, suggesting that their novelty and unexpectedness, are a source of enchantment, potentially boosting affective reading experience [25, 26, 76]. This is well in line with rating data from a different set of participants—we assume that processes underlying the effects of structural text features are largely common to different participant samples—who evaluated magical text passages as more surprise and reading-pleasure inducing than control stimuli.

Given the limited temporal resolution of fMRI and the complexity of our stimulus material tapping into the complex, holistic process of reading entire text passages, and the scarcity of meta-analysis data for more natural reading tasks that would constrain reverse inferences [78], we cannot exactly say whether activation in high-level associative cortices in our data is selectively more related to one or another of the above-mentioned mental processes. Please note that besides the theoretical contribution, Hutzler [78] offers a practical solution to the problem of reverse inference, i.e. a Bayesian procedure that quantifies the value to be placed on such inferences – based on meta-analyses. However, limitations of available meta-analyses make it currently impossible to apply this procedure to our study: The calculation of the predictive quality requires task-specific hit rate and task-specific false positive rate, requiring the pooled result of all studies used for the meta-analysis, as provided e.g., in [89]. However, none of the meta-analyses relevant to our research question provides complete results (peaks) of studies included. Furthermore, most meta-analyses include only task-specific true positive results, while task-specific false positive results are required for the calculation of the predictive quality. Nevertheless, reverse inference based on task-constrained meta-analyses is more likely to have higher predictive value, because these inference have higher task-specific hit rate. Thus, the activation pattern in our results is perhaps best considered as tentative evidence for a repeated mutual interaction of attention to (emotional) salience and world knowledge integration, accompanied by affective processes of surprise and pleasure, between amygdala and the fronto-parietal network across the whole reading process. Despite these limitations, we are confident that our data help motivate and constrain future research to further specify the relationship between the amygdala (as a salience detector) and the fronto-parietal network (as an uncertainty resolver) when reading fictional texts and contribute to improve procedures for inferring mental processes underlying complex reading activities from functional imaging data [78].

## References

[1] Nell V (1988) Lost in a Book: The Psychology of Reading for Pleasure. New Haven/London: Yale University Press

[2] Kringelbach ML, Vuust P, Geake J (2008) The pleasure of reading. Interdiscipl Sci Rev 33: 321–335.

[3] Schrott R, Jacobs AM (2011) Gehirn und Gedicht: Wie wir unsere Wirklichkeiten konstruieren (Brain and Poetry: How We Construct Our Realities). München, Germany: Hanser 10.1080/17437199.2011.587961

[4] Sharon T, Woolley JD (2004) Do monsters dream? Young children's understanding of the fantasy/reality distinction. Brit J Dev Psychol 22: 293–310.

[5] Subbotsky E (2012) Discrimination between fantastic and ordinary visual displays by children and adults. Open Behavioral Science Journal 6: 23–30.

[6] Subbotsky E, Hysted C, Jones N (2010) Watching films with magical content facilitates creativity in children. Percept Motor Skill 111: 261–277. 21058605 10.2466/04.09.11.PMS.111.4.261-277

[7] Van Dijk TA, Kintsch W (1983) Strategies of discourse comprehension. New York: Academic Press

[8] Schmalhofer F, Glavanov D (1986) Three components of understanding a programmer's manual: Verbatim, propositional, and situational representations. J Mem Lang 25: 279–294.

[9] Graesser AC, Millis KK, Zwaan RA (1997) Discourse comprehension. Annu Rev Psychol 48: 163–189. 15012477 10.1146/annurev.psych.48.1.163

[10] Zwaan RA, Radvansky GA (1998) Situation models in language comprehension and memory. Psychol Bull 123: 162–185. 9522683 10.1037/0033-2909.123.2.162

[11] Hagoort P (2005) On Broca, brain, and binding: a new framework. Trends Cogn Sci 9: 416–423. 16054419 10.1016/j.tics.2005.07.004

[12] Hagoort P, Hald L, Bastiaansen M, Petersson KM (2004) Integration of word meaning and world knowledge in language comprehension. Science 304: 438–441. 15031438 10.1126/science.1095455

[13] Menenti L, Petersson KM, Scheeringa R, Hagoort P (2008) When elephants fly: differential sensitivity of right and left inferior frontal gyri to discourse and world knowledge. J Cogn Neurosci 21: 2358–2368. 10.1162/jocn.2008.21163 19016600

[14] Forgacs B, Bohrn I, Baudewig J, Hofmann MJ, Pleh C, et al (2012) Neural correlates of combinatorial semantic processing of literal and figurative noun noun compound words. NeuroImage 63: 1432–1442. 10.1016/j.neuroimage.2012.07.029 22836179

[15] Bohrn IC, Altmann U, Jacobs AM (2012) Looking at the brains behind figurative language-A quantitative meta-analysis of neuroimaging studies on metaphor, idiom, and irony processing. Neuropsychologia 50: 2669–2683. 10.1016/j.neuropsychologia.2012.07.021 22824234

[16] Mar RA, Oatley K (2008) The function of fiction is the abstraction and simulation of social experience. Perspect Psychol Sci 3: 173–192. 26158934 10.1111/j.1745-6924.2008.00073.x

[17] Raposo A, Vicens L, Clithero JA, Dobbins IG, Huettel SA (2011) Contributions of frontopolar cortex to judgments about self, others and relations. Soc Cogn Affect Neurosci 6: 260–269. 10.1093/scan/nsq033 20478834 PMC3110426

[18] Altmann U, Bohrn IC, Lubrich O, Menninghaus W, Jacobs AM (2012) The power of emotional valence-from cognitive to affective processes in reading. Front Hum Neurosci 6: 192 10.3389/fnhum.2012.00192 22754519 PMC3385211

[19] Altmann U, Bohrn IC, Lubrich O, Menninghaus W, Jacobs AM (2014) Fact vs fiction—how paratextual information shapes our reading processes. Soc Cogn Affect Neurosci 9: 22–29. 10.1093/scan/nss098 22956671 PMC3871725

[20] Addis DR, Pan L, Vu MA, Laiser N, Schacter DL (2009) Constructive episodic simulation of the future and the past: distinct subsystems of a core brain network mediate imagining and remembering. Neuropsychologia 47: 2222–2238. 10.1016/j.neuropsychologia.2008.10.026 19041331

[21] Frith U, Frith CD (2003) Development and neurophysiology of mentalizing. Philos Trans R Soc Lond B Biol Sci 358: 459–473. 12689373 10.1098/rstb.2002.1218 PMC1693139

[22] Pourtois G, Schettino A, Vuilleumier P (2013) Brain mechanisms for emotional influences on perception and attention: what is magic and what is not. Biol Psychol 92: 492–512. 10.1016/j.biopsycho.2012.02.007 22373657

[23] Miall DS, Kuiken D (1994) Foregrounding, defamiliarization, and affect—Response to literary stories. Poetics 22: 389–407.

[24] Oatley K (1995) A taxonomy of the emotions of literary response and a theory of identification in fictional narrative. Poetics 23: 53–74.

[25] Jacobs AM (2011) Neurokognitive Poetik: Elemente eines neurokognitiven Modells des literarischen Lesens [Neurocognitive poetics: Elements of a neurocognitive model of literary reading]. In: Schrott R, Jacobs AM, editors. Gehirn und Gedicht: Wie wir unsere Wirklichkeiten konstruieren. Munich, Germany: Carl Hanser Verlag pp. 492–524.

[26] Jacobs AM (2014) Towards a Neurocognitive Poetics Model of literary reading. In: Willems RM, editor. Towards a Cognitive Neuroscience of Natural Language Use. Cambridge: Cambridge University Press pp. 135–159.

[27] Jacobs AM, Lüdtke J, Meyer-Sickendiek B (2013) Bausteine einer Neurokognitiven Poetik: Foregrounding/Backgrounding, lyrische Stimmung und ästhetisches Gefallen [Building blocks of Neurocognitive Poetics: Foregrounding/Backgrounding, lyric mood and aesthetic liking]. In: Meyer-Sickendiek B, Reents F, editors. Stimmung und Methode. Tübingen: Mohr Siebeck.

[28] Seeley WW, Menon V, Schatzberg AF, Keller J, Glover GH, et al (2007) Dissociable intrinsic connectivity networks for salience processing and executive control. J Neurosci 27: 2349–2356. 17329432 10.1523/JNEUROSCI.5587-06.2007 PMC2680293

[29] Lindquist KA, Wager TD, Kober H, Bliss-Moreau E, Barrett LF (2012) The brain basis of emotion: a meta-analytic review. Behav Brain Sci 35: 121–143. 10.1017/S0140525X11000446 22617651 PMC4329228

[30] Corbetta M, Shulman GL (2002) Control of goal-directed and stimulus-driven attention in the brain. Nat Rev Neurosci 3: 201–215. 11994752 10.1038/nrn755

[31] Corbetta M, Patel G, Shulman GL (2008) The reorienting system of the human brain: from environment to theory of mind. Neuron 58: 306–324. 10.1016/j.neuron.2008.04.017 18466742 PMC2441869

[32] Costafreda SG, Brammer MJ, David AS, Fu CH (2008) Predictors of amygdala activation during the processing of emotional stimuli: a meta-analysis of 385 PET and fMRI studies. Brain Res Rev 58: 57–70. 18076995 10.1016/j.brainresrev.2007.10.012

[33] Viviani R (2013) Emotion regulation, attention to emotion, and the ventral attentional network. Front Hum Neurosci 7: 746 10.3389/fnhum.2013.00746 24223546 PMC3819767

[34] Rowling JK (1997) Harry Potter and the Philosopher's Stone. London: Bloomsbury

[35] Rowling JK (1998) Harry Potter and the Chamber of Secrets. London: Bloomsbury

[36] Rowling JK (1999) Harry Potter and the Prisoner of Azkaban. London: Bloomsbury

[37] Rowling JK (2000) Harry Potter and the Goblet of Fire. London: Bloomsbury

[38] Rowling JK (2003) Harry Potter and the Order of the Phoenix. London: Bloomsbury

[39] Rowling JK (2005) Harry Potter and the Half Blood Prince. London: Bloomsbury

[40] Rowling JK (2007) Harry Potter and the Deathly Hallows. London: Bloomsbury 10.1093/jxb/erm028

[41] Hsu C-T, Jacobs AM, Conrad M (2015) Can Harry Potter still put a spell on us in a second language? An fMRI study on reading emotion-laden literature in late bilinguals. Cortex 63: 282–295. 10.1016/j.cortex.2014.09.002 25305809

[42] Hsu C-T, Conrad M, Jacobs AM (2014) Fiction feelings in Harry Potter: haemodynamic response in the mid-cingulate cortex correlates with immersive reading experience. NeuroReport 25: 1356–1361. 10.1097/WNR.0000000000000272 25304498

[43] Võ ML, Conrad M, Kuchinke L, Urton K, Hofmann MJ, et al (2009) The Berlin Affective Word List Reloaded (BAWL-R). Behav Res Methods 41: 534–538. 10.3758/BRM.41.2.534 19363195

[44] Schmidtke DS, Schröder T, Jacobs AM, Conrad M (2014) ANGST: Affective Norms for German Sentiment Terms derived from the Affective Norms for English Words. Behav Res Methods 46: 1108–1118. 10.3758/s13428-013-0426-y 24415407

[45] Ashburner J, Friston KJ (2005) Unified segmentation. NeuroImage 26: 839–851. 15955494 10.1016/j.neuroimage.2005.02.018

[46] Ashburner J (2007) A fast diffeomorphic image registration algorithm. NeuroImage 38: 95–113. 17761438 10.1016/j.neuroimage.2007.07.007

[47] Leiner DJ (2014) SoSci Survey. (2.4.00-i). http://www.soscisurvey.com

[48] Friston KJ, Holmes AP, Poline JB, Grasby PJ, Williams SC, et al (1995) Analysis of fMRI time-series revisited. NeuroImage 2: 45–53. 9343589 10.1006/nimg.1995.1007

[49] Bach DR, Schachinger H, Neuhoff JG, Esposito F, Di Salle F, et al (2008) Rising sound intensity: an intrinsic warning cue activating the amygdala. Cereb Cortex 18: 145–150. 17490992 10.1093/cercor/bhm040

[50] Ewbank MP, Fox E, Calder A (2010) The interaction between gaze and facial expression in the amygdala and extended amygdala is modulated by anxiety. Front Hum Neurosci 4 10.3389/fnhum.2010.00228 20661452 PMC2906373

[51] Jenison RL, Rangel A, Oya H, Kawasaki H, Howard MA (2011) Value encoding in single neurons in the human amygdala during decision making. J Neurosci 31: 331–338. 10.1523/JNEUROSCI.4461-10.2011 21209219 PMC3028386

[52] Phan KL, Wager T, Taylor SF, Liberzon I (2002) Functional neuroanatomy of emotion: a meta-analysis of emotion activation studies in PET and fMRI. NeuroImage 16: 331–348. 12030820 10.1006/nimg.2002.1087

[53] Murphy FC, Nimmo-Smith I, Lawrence AD (2003) Functional neuroanatomy of emotions: a meta-analysis. Cogn Affect Behav Neurosci 3: 207–233. 14672157 10.3758/cabn.3.3.207

[54] Ferstl EC, Rinck M, von Cramon DY (2005) Emotional and temporal aspects of situation model processing during text comprehension: an event-related fMRI study. J Cogn Neurosci 17: 724–739. 15904540 10.1162/0898929053747658

[55] Maldjian JA, Laurienti PJ, Kraft RA, Burdette JH (2003) An automated method for neuroanatomic and cytoarchitectonic atlas-based interrogation of fMRI data sets. NeuroImage 19: 1233–1239. 12880848 10.1016/s1053-8119(03)00169-1

[56] Lieberman MD, Cunningham WA (2009) Type I and Type II error concerns in fMRI research: re-balancing the scale. Soc Cogn Affect Neurosci 4: 423–428. 10.1093/scan/nsp052 20035017 PMC2799956

[57] Lancaster JL, Rainey LH, Summerlin JL, Freitas CS, Fox PT, et al (1997) Automated labeling of the human brain: a preliminary report on the development and evaluation of a forward-transform method. Hum Brain Mapp 5: 238–242. 10.1002/(SICI)1097-0193(1997)5:4&lt;238::AID-HBM6&gt;3.0.CO;2-4 20408222 PMC2860189

[58] Lancaster JL, Woldorff MG, Parsons LM, Liotti M, Freitas CS, et al (2000) Automated Talairach atlas labels for functional brain mapping. Hum Brain Mapp 10: 120–131. 10912591 10.1002/1097-0193(200007)10:3<120::AID-HBM30>3.0.CO;2-8 PMC6871915

[59] Turkeltaub PE, Eden GF, Jones KM, Zeffiro TA (2002) Meta-analysis of the functional neuroanatomy of single-word reading: method and validation. NeuroImage 16: 765–780. 12169260 10.1006/nimg.2002.1131

[60] Ferstl EC, Neumann J, Bogler C, von Cramon DY (2008) The extended language network: a meta-analysis of neuroimaging studies on text comprehension. Hum Brain Mapp 29: 581–593. 17557297 10.1002/hbm.20422 PMC2878642

[61] Binder JR, Desai RH, Graves WW, Conant LL (2009) Where is the semantic system? A critical review and meta-analysis of 120 functional neuroimaging studies. Cereb Cortex 19: 2767–2796. 10.1093/cercor/bhp055 19329570 PMC2774390

[62] Binder JR, Desai RH (2011) The neurobiology of semantic memory. Trends Cogn Sci 15: 527–536. 10.1016/j.tics.2011.10.001 22001867 PMC3350748

[63] Kuchinke L, Jacobs AM, Grubich C, Vo ML, Conrad M, et al (2005) Incidental effects of emotional valence in single word processing: an fMRI study. NeuroImage 28: 1022–1032. 16084739 10.1016/j.neuroimage.2005.06.050

[64] Bohrn IC, Altmann U, Lubrich O, Menninghaus W, Jacobs AM (2013) When we like what we know—a parametric fMRI analysis of beauty and familiarity. Brain Lang 124: 1–8. 10.1016/j.bandl.2012.10.003 23332807

[65] Cohen L, Lehericy S, Chochon F, Lemer C, Rivaud S, et al (2002) Language-specific tuning of visual cortex functional properties of the Visual Word Form Area. Brain 125: 1054–1069. 11960895 10.1093/brain/awf094

[66] Price CJ, Devlin JT (2003) The myth of the visual word form area. NeuroImage 19: 473–481. 12880781 10.1016/s1053-8119(03)00084-3

[67] Dehaene S, Cohen L (2011) The unique role of the visual word form area in reading. Trends Cogn Sci 15: 254–262. 10.1016/j.tics.2011.04.003 21592844

[68] Price CJ, Devlin JT (2011) The interactive account of ventral occipitotemporal contributions to reading. Trends Cogn Sci 15: 246–253. 10.1016/j.tics.2011.04.001 21549634 PMC3223525

[69] Harel A, Kravitz D, Baker CI (2013) Beyond perceptual expertise: revisiting the neural substrates of expert object recognition. Front Hum Neurosci 7: 885 10.3389/fnhum.2013.00885 24409134 PMC3873520

[70] Herbert C, Ethofer T, Anders S, Junghofer M, Wildgruber D, et al (2009) Amygdala activation during reading of emotional adjectives—an advantage for pleasant content. Soc Cogn Affect Neurosci 4: 35–49. 10.1093/scan/nsn027 19015080 PMC2656883

[71] Markowitsch HJ (1998) Differential contribution of right and left amygdala to affective information processing. Behav Neurol 11: 233–244. 11568425 10.1155/1999/180434

[72] Schirmer A, Kotz SA (2006) Beyond the right hemisphere: brain mechanisms mediating vocal emotional processing. Trends Cogn Sci 10: 24–30. 16321562 10.1016/j.tics.2005.11.009

[73] Sander D, Grafman J, Zalla T (2003) The human amygdala: an evolved system for relevance detection. Rev Neurosci 14: 303–316. 14640318 10.1515/revneuro.2003.14.4.303

[74] Phan KL, Wager TD, Taylor SF, Liberzon I (2004) Functional neuroimaging studies of human emotions. CNS Spectr 9: 258–266. 15048050 10.1017/s1092852900009196

[75] Citron FM, Goldberg AE (2014) Metaphorical sentences are more emotionally engaging than their literal counterparts. J Cogn Neurosci 26: 2585–2595. 10.1162/jocn_a_00654 24800628

[76] Bohrn IC, Altmann U, Lubrich O, Menninghaus W, Jacobs AM (2012) Old proverbs in new skins—an FMRI study on defamiliarization. Front Psychol 3: 204 10.3389/fpsyg.2012.00204 22783212 PMC3389387

[77] Ortony A, Turner TJ (1990) What's basic about basic emotions? Psychol Rev 97: 315–331. 1669960 10.1037/0033-295x.97.3.315

[78] Hutzler F (2014) Reverse inference is not a fallacy per se: Cognitive processes can be inferred from functional imaging data. NeuroImage 84: 1061–1069. 10.1016/j.neuroimage.2012.12.075 23313571

[79] Briesemeister BB, Kuchinke L, Jacobs AM (2011) Discrete emotion effects on lexical decision response times. PLoS One 6: e23743 10.1371/journal.pone.0023743 21887307 PMC3161062

[80] Briesemeister BB, Kuchinke L, Jacobs AM (2014) Emotion word recognition: Discrete information effects first, continuous later? Brain Res 1564: 62–71. 10.1016/j.brainres.2014.03.045 24713350

[81] Briesemeister BB, Kuchinke L, Jacobs AM, Braun M (2014) Emotions in reading: Dissociation of happiness and positivity. Cogn Affect Behav Neurosci. 10.3758/s13415-014-0327-2 25398299

[82] Russell JA (1980) A circumplex model of affect. J Pers Soc Psychol 39: 1161–1178.

[83] Russell JA (2003) Core affect and the psychological construction of emotion. Psychol Rev 110: 145–172. 12529060 10.1037/0033-295x.110.1.145

[84] Spreng RN, Mar RA, Kim AS (2009) The common neural basis of autobiographical memory, prospection, navigation, theory of mind, and the default mode: a quantitative meta-analysis. J Cogn Neurosci 21: 489–510. 10.1162/jocn.2008.21029 18510452

[85] Seghier ML (2013) The angular gyrus: multiple functions and multiple subdivisions. Neuroscientist 19: 43–61. 10.1177/1073858412440596 22547530 PMC4107834

[86] Ma WJ, Husain M, Bays PM (2014) Changing concepts of working memory. Nat Neurosci 17: 347–356. 10.1038/nn.3655 24569831 PMC4159388

[87] Buckner RL, Andrews-Hanna JR, Schacter DL (2008) The brain's default network: anatomy, function, and relevance to disease. Ann N Y Acad Sci 1124: 1–38. 10.1196/annals.1440.011 18400922

[88] Mar RA (2011) The neural bases of social cognition and story comprehension. Annu Rev Psychol 62: 103–134. 10.1146/annurev-psych-120709-145406 21126178

[89] Vigneau M, Beaucousin V, Hervé PY, Duffau H, Crivello F, et al (2006) Meta-analyzing left hemisphere language areas: Phonology, semantics, and sentence processing. NeuroImage 30: 1414–1432. 16413796 10.1016/j.neuroimage.2005.11.002

